# The long non-coding RNA NNT-AS1 promotes clear cell renal cell carcinoma progression via regulation of the miR-137/ Y-box binding protein 1 axis

**DOI:** 10.1080/21655979.2021.1992330

**Published:** 2021-11-25

**Authors:** Yadi Zhou, Zhenghao Zhang, Mingyi Wo, Wenfang Xu

**Affiliations:** a Clinical Laboratory, Affiliated Hospital of Shaoxing University, Shaoxing, China; b Department of Clinical Laboratory, Zhejiang Provincial People’s Hospital, Hangzhou Medical College, Hangzhou, China

**Keywords:** NNT-AS1, clear cell renal cell carcinoma, miR-137, YBX-1

## Abstract

Long noncoding RNAs (lncRNAs) have been implicated in the progression of malignant tumors, including in clear cell renal cell carcinoma (ccRCC). However, the function and the specific mechanism of lncRNA nicotinamide nucleotide transhydrogenase antisense RNA 1 (NNT-AS1) in ccRCC remains unknown. Thus, this study explored the role of NNT-AS1 in ccRCC. We evaluated NNT-AS1 expression in ccRCC specimens. Next, CCK-8 and Transwell assays were used to evaluate cell proliferation and metastatic abilities. The interaction between miR-137 and NNT-AS1 or Y-box binding protein 1 (YBX-1) was confirmed using a dual luciferase reporter assay. The results showed that NNT-AS1 was significantly upregulated in ccRCC specimens compared with normal tissues. Inhibition of NNT-AS1 restrained ccRCC proliferation and metastasis. Mechanistically, NNT-AS1 acted as a competitive endogenous RNA to sponge miR-137, which depressed ccRCC cells proliferation and metastasis. Moreover, with the use of bioinformatics analysis, the famous oncogene YBX-1 was selected as the potential target of miR-137. Luciferase assay also confirmed the interaction between miR-137 and YBX-1. Further functional studies demonstrated that the inhibition effect of NNT-AS1 knockdown on ccRCC carcinogenesis could be partially reversed by overexpression of YBX-1, suggesting that NNT-AS1 promotes ccRCC progression through the miR-137/YBX-1 pathway. In summary, these findings indicate that NNT-AS1 promotes ccRCC progression via the miR-137/YBX-1 pathway, which may provide a promising therapeutic target for renal cell carcinoma.

## Introduction:

Renal cell carcinoma (RCC) is among the 10 most prevalent cancers worldwide [1]. RCC can be histologically categorized into clear cell RCC, chromophobe RCC (chRCC) or papillary RCC (pRCC) [2–4]. ccRCC is one of the main subtypes of RCC, accounting for 75% of all RCC cases. Although surgical or ablative strategies can successfully treat ccRCC patients, nearly one-third of patients are diagnosed with metastases [5]. Therefore, identifying the specific molecular mechanisms involved in the progression of ccRCC will contribute to ccRCC treatment.

In the human genome, only 2% of DNA can be transcribed to mRNA, while the remaining 98% can be transcribed as noncoding RNA [6]. Among these noncoding RNAs, RNAs with lengths greater than 200 nucleotides are classified as long noncoding RNAs (lncRNAs) [7]. Over the past 20 years, many studies have uncovered that lncRNAs participate in numerous physiological or pathological processes, including stem cell maintenance and differentiation, cardiovascular diseases and carcinogenesis [8,9]. In particular, lncRNAs can exert their functions via regulation of related genes at the epigenetic, transcriptional, or post-transcriptional levels. Recently, lncRNAs have been confirmed to play important roles in ccRCC, including DMDRMR [10], Lnc-TSI [11], MRCCAT1 [12] and CRNDE [13]. Recently, the lncRNA NNT-AS1 has been reported to be dysregulated in several solid tumors, including lung cancer [14], glioma [15] and cervical cancer [16]. Ma et al. reported that NNT-AS1 modulates the expression of FOXM1 by serving as a sponge of miR-22, thus contributing to lung squamous cell carcinoma progression [14]. Another study also revealed that NNT-AS1 promotes cervical cancer resistance to cisplatin treatment via the miR-186/HMGB1 axis [16]. However, the function of NNT-AS1 in ccRCC was little reported. Thus, it is meaningful to uncover the vital role of NNT-AS1 in ccRCC.

MicroRNAs (miRNAs) are defined as small non-coding RNA molecules that regulate gene expression through binding to the mRNA of target genes [17]. miRNAs can regulate the expression of many mRNAs and thus are involved in many physiological and pathological pathways [18,19]. Various miRNAs have been found to be differentially expressed in ccRCC, and miRNAs can also serve as diagnostic and prognostic biomarkers for ccRCC. Recently, lncRNAs have been found to contain many miRNA-binding sites that can sponge miRNAs to indirectly regulate mRNA expression. The important role of lncRNA–miRNA–mRNA axis has also been confirmed in ccRCC [20,21]. Therefore, clarification of the lncRNA-miRNA-mRNA regulatory axis may facilitate the prevention, diagnosis and treatment of ccRCC.

To this end, in this study, the biological role of NNT-AS1 in ccRCC was explored. The results revealed that NNT-AS1 was significantly upregulated in ccRCC specimens compared with normal tissues. Thus, it was hypothesized that NNT-AS1 may function as an oncogene in the ccRCC progression. Mechanically, NNT-AS1 was found to serve as a ceRNA to sponge miR-137, which specifically targeted the oncogene YBX-1. Ultimately, the goal of this study is to explore whether NNT-AS1 promotes ccRCC progression via the miR-137/YBX-1 axis. These findings provide a promising therapeutic target for renal cell carcinoma.

## Material and methods

### Cell culture

Human ccRCC cell lines (786-O, OS-RC-2, A498 and Caki-1) and HEK-293 T cell lines were purchased from American Type Culture Collection (ATCC) (Manassas, VA). The cells were cultured in RPMI-1640 harboring 10% fetal bovine serum at 37°C with 5% CO_2_ [22].

### Sample collection

A total of 40 surgical ccRCC specimens and matched normal tissues were collected at the Affiliated Hospital of Shaoxing University from February 2017 to May 2020. Patients who received chemotherapy or radiotherapy were excluded. The experimental protocols were approved by the Ethics Committee of the Affiliated Hospital of Shaoxing University.

### Cell transfection

siRNAs targeting NNT-AS1, miR-137 mimics and inhibitors were obtained from RiboBio (Guangzhou, China). For siRNA and miR-137 mimics or inhibitors transfection, ccRCC cells were plated into the six-well plate. Lipofectamine RNAiMAX (Invitrogen) was used as a transfection agent according to the provided protocols. The NNT-AS1 and YBX-1 overexpression plasmids were obtained from GenePharma (Shanghai, China), and transfection was performed with Lipofectamine 3000 (Invitrogen, USA) according to the provided protocols.

### Luciferase reporter assay

The detailed methods were reported previously [23]. Briefly, 2 × 10^5^ of HEK-293 T cells were seeded in a 48-well plate individually and co-transfected with pGL3-NNT-AS1 or pGL3-YBX-1 luciferase plasmids as well as pGL3 control vector. Subsequently, miR-137 mimics or negative control were used to transfect the ccRCC cells. The luciferase activity was measured using a dual-luciferase reporter kit (Vazyme, China) according to the manufacturer’s protocols.

### RNA extraction and qRT-PCR

Total RNA was extracted using TRIzol reagent (Invitrogen, Carlsbad, CA, USA). cDNA was synthesized using the GoScript Reverse Transcription (RT) system (Promega, Madison, WI, USA). RNA levels were measured by qRT-PCR on Roche Light Cycler 480 using the GoTaq qPCR Master Mix system (Promega, Madison, WI, USA). The expression of lncRNA and mRNA was normalized with GAPDH. The primer sequences are listed in the supplementary material.

### CCK-8 assay

2 × 10^3^ cells ccRCC cells were plated into 96-well plate and incubated for 0 h, 24 h, 48 h, 72 h, and 96 h. Then, 10 μL of CCK-8 reagent (Dojindo, Japan) was added into cells, and the cells were incubated at 37°C for another 1 hour. Finally, the absorbance was measured at 450 nm.

### Transwell assay

For the cell invasion assays, 6 × 10^5^ ccRCC cells were placed into the upper chamber and were covered with Matrigel and a complete medium was added into the lower chamber for 48 hours. For the cell migration assays, 2 × 10^5^ cells were placed into the upper chamber and complete medium was added into the lower chamber for 24 hours. Finally, all cells were fixed with paraformaldehyde and stained with crystal violet.

### RNA immunoprecipitation (RIP) assay

The detailed methods were reported previously [24]. Briefly, RNA immunoprecipitation assays were performed using Magna RIP Kit (Millipore, USA) according to the manufacturer’s methods. Immunoprecipitated RNAs were purified and analyzed by RT-qPCR [25].

### Western blotting

Total proteins were extracted from ccRCC cells using RIPA lysis buffer (Beyotime, Shanghai, China). The proteins were separated by SDS-PAGE on 10% gels and transferred to PVDF membranes (Millipore, Billerica, USA). After blocking with 5% BSA, the membrane was incubated with primary antibodies overnight at 4°C. The next day, the membrane was washed, followed by incubation with a secondary antibody. Finally, the ECL was used to visualize the protein signals [25].

### Animal experiments

OSRC-2 cells were stably transfected with control and si-NNT-AS1. About 2 × 10^6^ stably transfected OSRC-2 cells were subcutaneously injected into the armpit regions of the BALB/c nude mice (4 weeks, female). Tumor volumes were measured every 5 days. After 3 weeks, mice were sacrificed, and the volume of tumors was measured.

### Statistical analysis

Data are presented as mean ± SEM. The correlation of the expression between two groups was identified by Spearman’s correlation analysis. Student’s t-test and ANOVA were used to calculate the differences between the groups. All statistical analyses were performed using GraphPad Prism software. We considered results statistically significant when *P* < 0.05.

## Results:

The goal of this study was to explore the specific role of the lncRNA NNT-AS1 in ccRCC. The results indicated that NNT-AS1 was upregulated in both ccRCC cell lines and ccRCC specimens, suggesting that it could be a promising biomarker for the diagnosis of ccRCC. Proliferation, transwell assay and xenograft mice model assay showed that NNT-AS1 promoted ccRCC cell proliferation and metastasis in vitro and in vivo. Moreover, the mechanism assays showed that NNT-AS1 promoted ccRCC progression through the miR-137/YBX-1 axis.

### lncRNA NNT-AS1 expression is elevated in ccRCC tissues and cell lines

**Figure 1.**
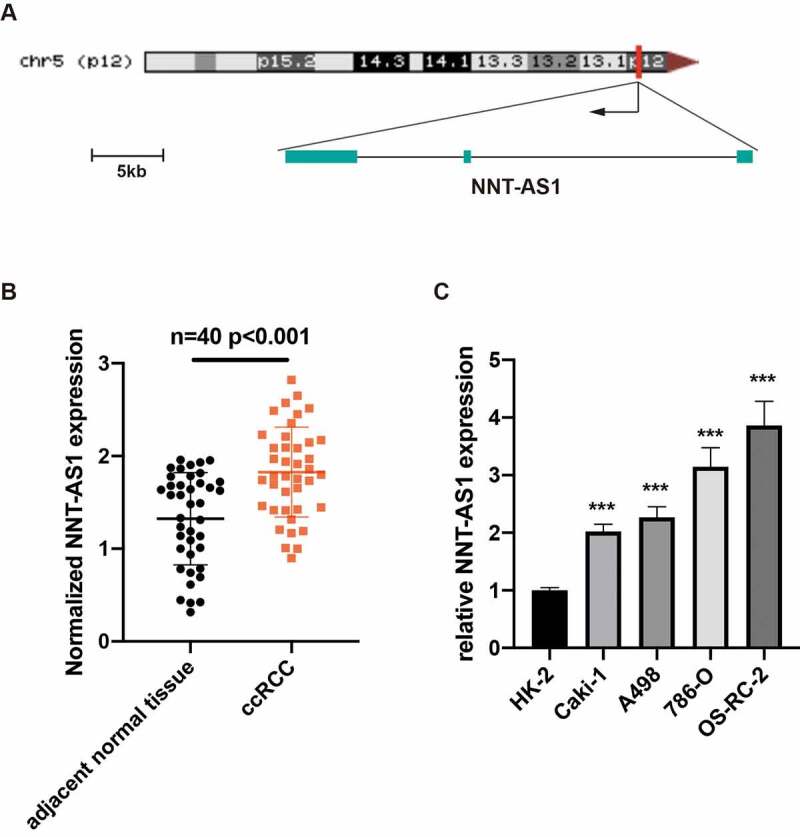
LncRNA NNT-AS1 expression is elevated in ccRCC tissues and cell lines. **A** Schematic representation of NNT-AS1. **B** NNT-AS1 expression level in ccRCC tissues and matched normal tissues by qRT-PCR. **C** NNT-AS1 expression level in ccRCC cell lines and normal renal cell line HK-2 by qRT-PCR. ***P < 0.001

NNT-AS1 is a newly reported lncRNA that is located in chr5:43,571,594–43,603,138; the transcript is 4803 nucleotides (nt) in length (Figure 1a). To verify the NNT-AS1 expression in ccRCC tissues and cell lines, qRT-PCR was performed. The expression of NNT-AS1 was significantly elevated in ccRCC tissues compared with adjacent normal tissues (Figure 1b). Furthermore, the expression level of NNT-AS1 was increased in the Caki-1, OS-RC-2, A498 and 786-O ccRCC cell lines compared with normal renal cell line HK-2 (Figure 1c). Thus, these results presented that NNT-AS1 may be correlated with ccRCC progression.

### Silencing of NNT-AS1 restrains ccRCC proliferation and metastasis

**Figure 2.**
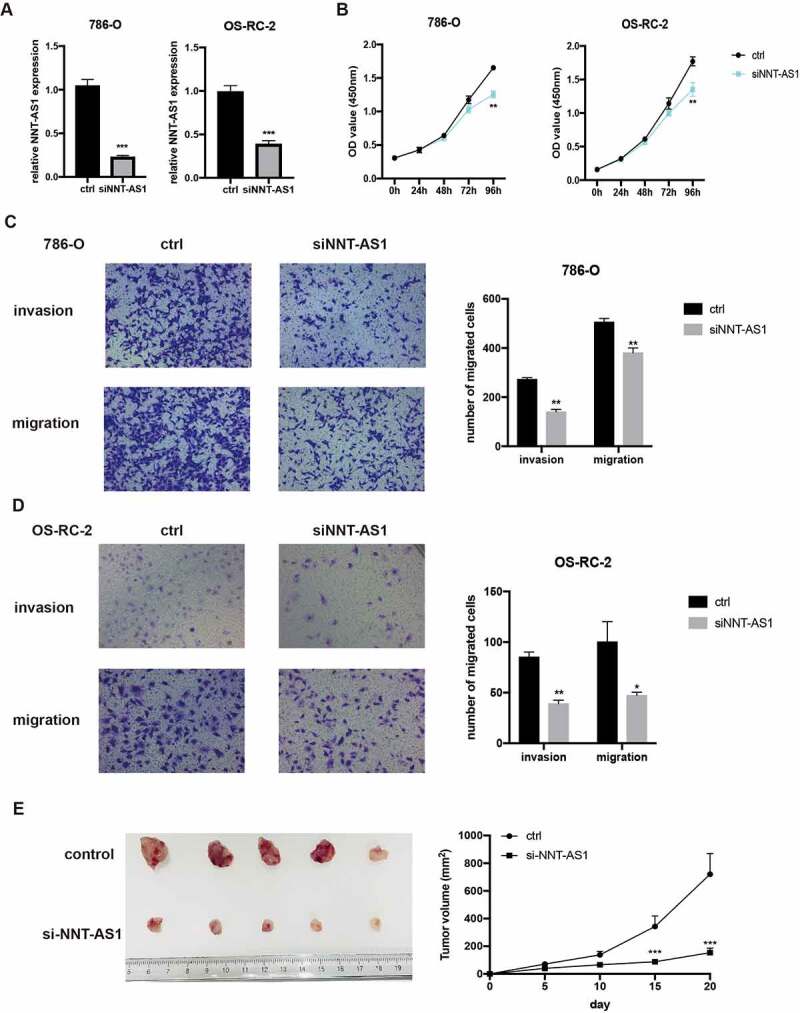
Silencing of NNT-AS1 restrains ccRCC proliferation and metastasis. **A** Verification of NNT-AS1 siRNA efficiency by qRT-PCR analysis. **B** Effect of NNT-AS1 knockdown on ccRCC cells proliferation. **C and D** Effect of NNT-AS1 knockdown on ccRCC metastasis. **E** Effect of NNT-AS1 knockdown in in vivo xenografted animal model. *P < 0.05, ** P < 0.01, ***P < 0.001

To ascertain the function of NNT-AS1 in ccRCC, we knocked down or overexpressed NNT-AS1 in ccRCC cells. First, qRT-PCR was used to measure the knockdown efficiency. The results revealed that NNT-AS1 was evidently decreased in ccRCC cells after transfection with NNT-AS1 siRNA (Figure 2a). Then, CCK8 assay results revealed that the ability of proliferation in ccRCC cells was inhibited after knockdown of NNT-AS1 (Figure 2b). To measure the metastasis ability of NNT-AS1, transwell assay was conducted and the results revealed that silencing of NNT-AS1 significantly inhibited the ccRCC cell metastasis ability compared to normal treatment (Figure 2c and Figure 2d). In vivo xenografted animal model also demonstrated that the volume of tumor generated by si-NNT-AS1 cells was significantly smaller than that from control cells (Figure 2e). These findings indicate that knockdown of NNT-AS1 contributes to the inhibition of ccRCC progression both in vitro and in vivo.

### Overexpression of NNT-AS1 promotes ccRCC proliferation and metastasis

**Figure 3.**
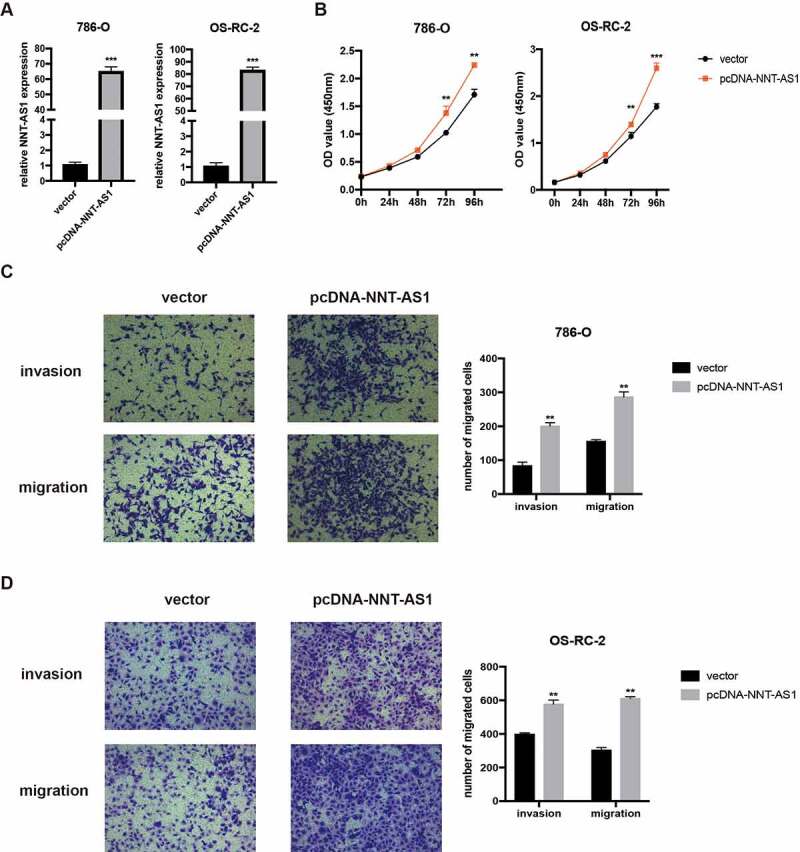
Overexpression of NNT-AS1 promotes ccRCC proliferation and metastasis. **A** Verification of the NNT-AS1 plasmids overexpression efficiency by qRT-PCR analysis. **B** Effect of NNT-AS1 overexpression on ccRCC cells proliferation. **C and D** Effect of NNT-AS1 overexpression on ccRCC metastasis. ** P < 0.01, ***P < 0.001

We then constructed the NNT-AS1 overexpression plasmid to explore the role of NNT-AS1 in ccRCC. First, qRT-PCR assay was used to verify the overexpression efficiency (Figure 3a). Next, CCK-8 assay confirmed that overexpression of NNT-AS1 enhanced the proliferation ability of ccRCC cells (Figure 3b). Moreover, overexpression of NNT-AS1 strengthened the metastatic ability of ccRCC cells (Figure 3c,d). These results suggest that overexpression of NNT-AS1 promotes ccRCC progression.

### NNT-AS1 can directly bind to miR-137 in ccRCC cells

**Figure 4.**
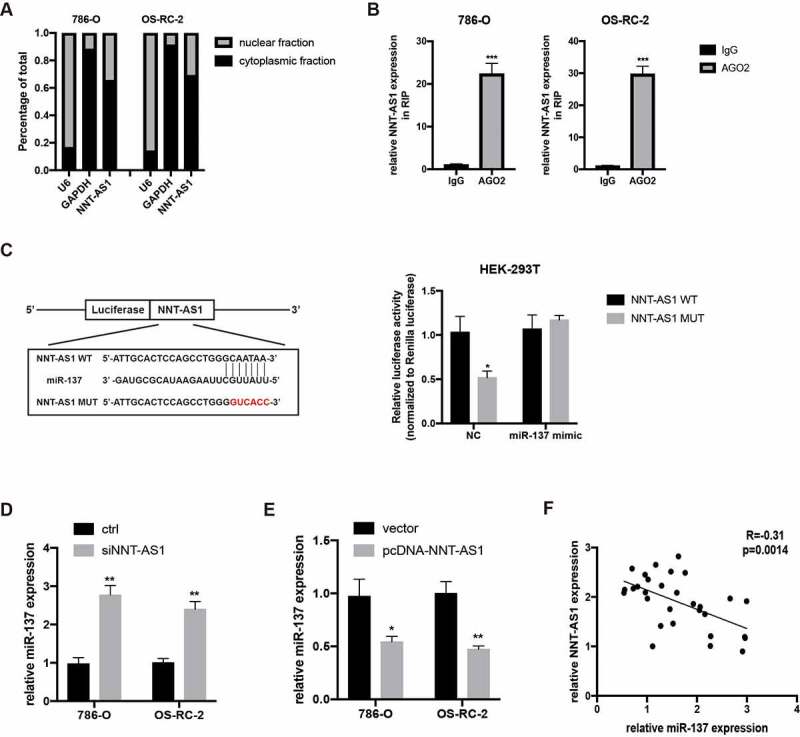
NNT-AS1 can directly bind to miR-137. **A** qRT-PCR analysis of nuclear and cytoplasmic RNA fraction in ccRCC cells. **B** AGO-2 RNA-IP assay followed qRT-PCR analysis showing binding capacity between NNT-AS1 and AGO2. **C** Left, the predicted binding site of WT NNT-AS1 or MUT NNT-AS1 with miR-137; right, luciferase activity was detected in HEK-293 T cells. **D and E** Effect of the NNT-AS1 knockdown or overexpression on miR-137 expression. **F** Correlation analysis between NNT-AS1 and miR-137. *P < 0.05, ** P < 0.01, ***P < 0.001

To explore the specific mechanism underlying the regulation of ccRCC cell progression by NNT-AS1, first, the subcellular distribution of NNT-AS1 in ccRCC cells was examined. The result revealed that NNT-AS1 exists both in the nucleus and cytoplasm, with the greatest abundance in the cytoplasm (Figure 4a), suggesting that NNT-AS1 may exert its function at the post-transcriptional level. Next, AGO2 RNA-IP assay was conducted, and the results confirmed that NNT-AS1 directly binds to AGO2 (Figure 4b), suggesting that NNT-AS1 may serve as a ceRNA to sponge miRNAs. To verify this hypothesis, an online bioinformatics database (DIANA Tools) was used, and miR-137 was identified as a candidate miRNA that can bind to NNT-AS1. A dual-luciferase reporter assay was then performed to confirm this prediction. As shown in Figure 4c, the luciferase activity decreased in HEK-293 T cells when transfected with WT-NNT-AS1 and miR-137 mimics, while there was no difference in the luciferase activity when HEK-293 T cells were transfected with MUT-NNT-AS1 or miR-NC. Next, miR-137 expression was evaluated with knockdown or overexpression of NNT-AS1. Notably, the knockdown of NNT-AS1 significantly elevated miR-137 expression, while the overexpression of NNT-AS1 decreased miR-137 expression (Figure 4d,e). Moreover, qRT-PCR assay showed an inverse correlation between NNT-AS1 expression and miR-137 expression in ccRCC tissues (figure 4f). These results indicated that NNT-AS1 directly binds to miR-137.

### NNT-AS1 activity is partially mediated through negative regulation of miR-137

**Figure 5.**
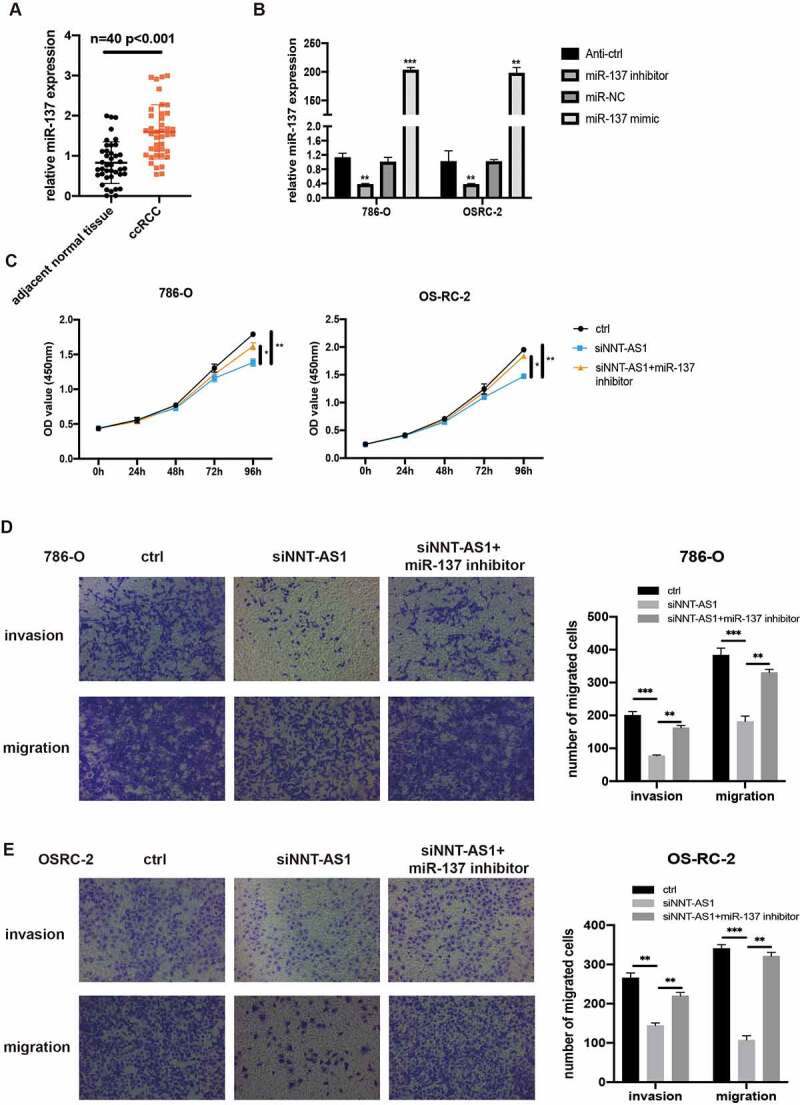
NNT-AS1 activity is partially mediated through negatively regulation of miR-137. **A** miR-137 expression level in ccRCC tissues and matched normal tissues. **B** Verification of the effect of miR-137 mimics or inhibitors by qRT-PCR analysis. **C** Effect of siNNT-AS1 or siNNT-AS1 plus miR-137 inhibitors on ccRCC cells proliferation. **D and E** Effect of siNNT-AS1 or siNNT-AS1 plus miR-137 inhibitors on ccRCC cells metastasis. *P < 0.05, ** P < 0.01, ***P < 0.001

To illustrate the hidden function and mechanism of miR-137, first, miR-137 expression was examined in 40 paired ccRCC tissues. qRT-PCR assay revealed that miR-137 was overexpressed in the ccRCC tissues compared with the matched normal tissues (Figure 5a). We then investigate the biological function of miR-137 in ccRCC by transfecting ccRCC cells with miR-137 inhibitors or mimics. qRT-PCR assay was first used to examine the overexpression or knockdown efficiency of miR-137 mimics or miR-137 inhibitors in ccRCC cells (Figure 5b). Then, CCK-8 assay revealed that the inhibited proliferation ability of ccRCC cells which is induced by the knockdown of NNT-AS1 expression can be rescued, at least partially, by co-transfection with miR-137 inhibitors (Figure 5c). Similarly, transwell assay suggested that the decreased number of metastatic ccRCC cells induced by transfection with siNNT-AS1 can be reversed by co-transfection with miR-137 inhibitors (Figures 5d, e). These findings indicate that NNT-AS1 promotes ccRCC progression through suppression of miR-137 activity.

### NNT-AS1 indirectly regulates YBX-1 expression through competitively binding with miR-137

**Figure 6.**
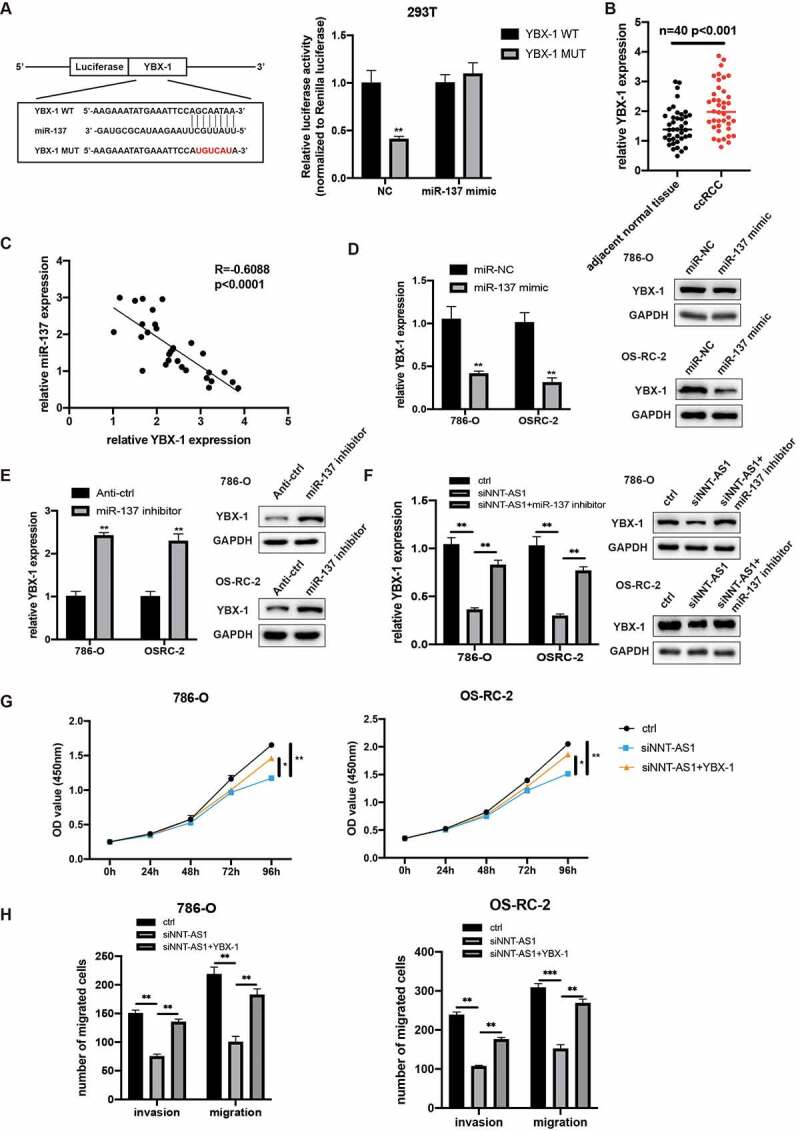
NNT-AS1 indirectly regulates YBX-1 expression via competitively binding with miR-137. **A** Left, the predicted binding site of miR-137 with wild type 3ʹ UTR of YBX-1 or mutant YBX-1; right, luciferase activity was detected in HEK-293 T cells. **B** miR-137 expression level in ccRCC tissues and matched normal tissues. **C** Correlation analysis showed a negatively correlation between YBX-1 and miR-137. **D** Effect of miR-137 mimic on YBX-1 expression. **E** effect of miR-137 inhibitors on YBX-1 expression. **E** Effect of siNNT-AS1 and siNNT-AS1 plus miR-137 inhibitors on YBX-1 expression. **G** Effect of siNNT-AS1 and siNNT-AS1 plus YBX-1 overexpression on ccRCC cells proliferation. **H** Effect of siNNT-AS1 and siNNT-AS1 plus YBX-1 overexpression on ccRCC cells metastasis. *P < 0.05, ** P < 0.01, ***P < 0.001

To explore the specific mechanism underlying the role of NNT-AS/miR-137 in ccRCC, miRDB and TargetScan were used to predict the potential target gene. YBX-1, a well-known oncogene, was identified to directly bind to miR-137. Next, the dual-luciferase reporter assay revealed that the co-transfection of miR-137 mimics and YBX-1 3ʹUTR reporter vector reduced the luciferase activity, while the co-transfection of miR-137 mimics and YBX-1 3ʹUTR MUT reporter vector has no differences in luciferase activity in HEK-293 T cells (Figure 6a). Then, YBX-1 expression was evaluated in 40 paired ccRCC tissues, and the qRT-PCR assay suggested that YBX-1 was overexpressed in ccRCC tissues (Figure 6b). Furthermore, the YBX-1 expression demonstrated an inverse correlation with miR-137 in ccRCC specimens (Figure 6c). Next, we evaluate the YBX-1 expression when transfected ccRCC cells with miR-137 inhibitors and mimics. As expected, qRT-PCR and western blot assay demonstrated that YBX-1 expression was upregulated with knockdown of miR-137, while YBX-1 expression was diminished with overexpression of miR-137 (Figures 6d, e). As NNT-AS1 could sponge miR-137, we next determined whether NNT-AS1 could regulate the expression of YBX-1 by inhibiting the activity of miR-137. qRT-PCR and western blot assay revealed that knockdown of NNT-AS1 decreased the mRNA and protein levels of YBX-1, while co-transfection of siNNT-AS1 and miR-137 inhibitors rescued the inhibition effect of siNNT-AS1 in ccRCC cells (figure 6f). Moreover, CCK-8 assay revealed that inhibition of NNT-AS1 reduced the proliferation ability of ccRCC cells, and this effect could be rescued by co-transfection with YBX-1 plasmids (Figure 6g). Meanwhile, transwell assay demonstrated that overexpression of YBX-1 could rescue the inhibition effect of siNNT-AS1 (Figure 6h). These results suggested that NNT-AS1 regulates YBX-1 expression via sponging of miR-137, and NNT-AS1 exerts its oncogenic roles through the regulation of YBX-1 expression.

## Discussion:

At present, with the development of diagnostic technology, surgical operation methods, and chemotherapy, the mortality of ccRCC has decreased. However, the overall survival rate of ccRCC is still unsatisfactory, especially in metastatic ccRCC patients [3]. Hence, understanding of the detailed molecular mechanism of ccRCC is valuable for ccRCC diagnosis and treatment. Recent evidence suggests that lncRNAs contribute to various biological processes, including cell proliferation, tumor metastasis, drug resistance and angiogenesis and so on [22,23]. In ccRCC, lncRNA LINC02747 can sponge miR-603, and then activate TFE3, with the latter promoting the proliferation of ccRCC [24]. DARS-AS1, a newly found lncRNA, can also promote ccRCC progression via the miR-194-5p/DARS pathway [25]. lncRNA NNT-AS1, a well-known lncRNA, has been found to be upregulated in various cancers, including lung cancer, glioma and cervical cancer [14–16]. In this study, we identified that NNT-AS1 was upregulated in ccRCC tissues compared to matched normal tissues. CCK-8 and transwell assays showed that silencing of NNT-AS1 significantly restrained ccRCC proliferation and metastasis, while overexpression of NNT-AS1 promoted ccRCC proliferation and invasion. These data suggested that NNT-AS1 exerted an oncogenic role in ccRCC progression. Then, the detailed mechanism of NNT-AS1 in ccRCC progression was explored. Cellular distribution analysis revealed that NNT-AS1 was mostly distributed in the cytoplasm, suggesting that NNT-AS1 may exert its role at the post-transcriptional level. Furthermore, AGO2 RNA-IP assay confirmed that NNT-AS1 could specifically bind to AGO2, a member of Argonaute family that plays the vital role in small RNA-induced-post-transcriptional gene silencing [26–30]. This finding suggests that NNT-AS1 could serve as a competitive endogenous RNA by binding to miRNAs to exert its biological function. Using an online bioinformatics database, miR-137 was selected as the downstream target of NNT-AS1. Luciferase reporter assay also confirmed an interaction between the NNT-AS1 and miR-137. Moreover, the silencing of NNT-AS1 increased the expression of miR-137, while overexpression of NNT-AS1 decreased the miR-137 expression. Then, we detected miR-137 expression in ccRCC tissues. Correlation analysis revealed that miR-137 and NNT-AS1 were inversely correlated in ccRCC tissues. These data indicate that NNT-AS1 may exert its function via modulation of miR-137 expression.

miRNAs are small noncoding RNAs of 17–25 nucleotides. miRNAs exert their roles mainly through recognizing the complementary target sites in the 3ʹUTR region of mRNA. They then inhibit the translation and stability of mRNAs. Dysregulated miRNAs exert their function through alteration of the expression of oncogenic or tumor-suppressive genes, which, in turn, promotes or inhibits tumor progression. For example, miR-124 and miR-203 have been reported to synergistically inhibit the EMT pathway through the regulation of ZEB2 expression in ccRCC [31]. Another group also revealed that miR-122 can promote ccRCC progression through targeting occlusion [32]. These two examples suggest that miRNAs promote or inhibit ccRCC progression through targeting oncogenic or tumor-suppressive genes. miR-137 has been found to suppress tumor progression, metastasis in melanoma, prostate cancer and ccRCC [33–35]. Luo et al. reported that miR-137 regulates ferroptosis via targeting SLC1A5, leading to reduced accumulation of glutamine uptake and malondialdehyde (MDA) [33]. In the current study, CCK-8 and transwell assays were applied to explore the role of miR-137 in ccRCC progression. The results showed that miR-137 restrained ccRCC progression in vitro, indicating that miR-137 is a tumor-suppressive miRNA. By using TargetScan and miRDB database prediction and dual-luciferase reporter assay verification, YBX-1 was identified as a downstream target of miR-137. Repression of miR-137 evidently increased the expression of YBX-1 both at the mRNA and protein level, while knockdown of NNT-AS1 produced the opposite effect. Then, a rescue assay was performed to explore whether NNT-AS1 regulated YBX-1 expression through miR-137. qRT-PCR and western blot assay confirmed this hypothesis. Moreover, CCK-8 and transwell assay also confirmed that the inhibition effect of NNT-AS1 in ccRCC progression could be reversed by overexpression of YBX-1. These results demonstrate that NNT-AS1 contributes to ccRCC progression through the miR-137/YBX-1 axis.

## Conclusion:

In this study, NNT-AS1 was proved to be overexpressed in ccRCC tissues and cell lines. Functionally, NNT-AS1 promoted ccRCC cells proliferation and metastasis. Mechanistically, NNT-AS1 enhanced the ccRCC malignancy through regulation of the miR-137/YBX-1 pathway. Therefore, inhibition of NNT-AS1 could be a promising therapeutic target for the treatment of ccRCC patients.

## Supplementary Material

Supplemental Material
