# Supplementary material for: The long non-coding RNA NNT-AS1 promotes clear cell renal cell carcinoma progression via regulation of the miR-137/ Y-box binding protein 1 axis
Source: Bioengineered. 2021 Nov 25;12(1):8994–9005. doi: 10.1080/21655979.2021.1992330 (PMC8806961; doi:10.1080/21655979.2021.1992330)
Supplement: Supplemental Material [file KBIE_A_1992330_SM8935.docx]

**Primers used for quantitative Real Time-PCR in this study.**

| Gene Symbol | Forward primer (5’→3’) | Reverse primer (5’→3’) | |
| --- | --- | --- | --- |
| NNT-AS1 | CTGGAATCCC TGCTACTCAGGA | GCCATGTGATATGCCTGCTC | |
| YBX-1 | GGGGACAAGAAGGTCATCGC | CGAAGGTACTTCCTGGGGTTA | |
| GAPDH | CAAGGTCATCCATGACAACTTTG | GTCCACCACCCTGTTGCTGTAG | |
| Primers used for stem-loop RT-PCR of miRNAs | | | |
| Gene Symbol | Stem-loop Reverse transcription primers (5’→3’) | Forward primer (5’→3’) | Reverse primer (5’→3’) |
| miR-137 | GTCGTATCCAGTGCAGGGTCCGAGGTATTCGCACTGGATACGACATTATC | GCGCGCTTATTGCTTAAGAATAC | GTGCAGGGTCCGAGGT |
| U6 | AACGCTTCACGAATTTGCGT | CTCGCTTCGGCAGCACA | AACGCTTCACGAATTTGCGT |

**Antibody used in this study**

| Antibody | company |
| --- | --- |
| GAPDH | Abcam (ab9485) |
| YBX1 | Abcam(ab76149) |
